# Postpartum Ischemic Stroke As the First Manifestation of Late-Onset Preeclampsia: A Case Report

**DOI:** 10.7759/cureus.105545

**Published:** 2026-03-20

**Authors:** Iliass Benaini, Meriam Azirar, Amine Bensaid, Mohamed Zakaria Bouayed, Safaa Kachmar, Samia Berrichi, Houssam Bkiyar

**Affiliations:** 1 Intensive Care and Anesthesia Department, Mohammed VI University Hospital, Oujda, MAR; 2 Simulation Center, Laboratory of Anatomy, Microsurgery, Experimental Surgery and Medical Simulation (LAMESMS), Faculty of Medicine and Pharmacy, Mohammed I University, Oujda, MAR

**Keywords:** ischemic stroke, mri, postpartum, preeclampsia, pregnancy

## Abstract

Preeclampsia is a severe pregnancy hypertensive disorder characterized by arterial hypertension and multiorgan involvement, most frequently affecting the renal, hepatic, and neurological systems. While its typical manifestations include headaches, visual disturbances, and/or epigastric pain, atypical presentations may occur and may lead to life-threatening complications.

We report the case of a 33-year-old multiparous woman who developed sudden left-sided hemiparesis, hemisensory loss, homonymous hemianopia, and rightward conjugate gaze deviation within 24 hours after a cesarean delivery. Blood pressure was 169/103 mmHg. Laboratory tests revealed moderate anemia, elevated LDH, low haptoglobin, and significant proteinuria (4.84 g/24h). Brain MRI demonstrated ischemic lesions in the right fronto-parieto-insular cortico-subcortical regions, consistent with a subacute infarction. The diagnosis of preeclampsia complicated by ischemic stroke was established. The patient was treated with acetylsalicylic acid, levetiracetam, and antihypertensive therapy (methyldopa and nicardipine). She progressively improved and achieved full neurological recovery within six weeks. Prophylaxis with low-dose aspirin and low molecular weight heparin was recommended for future pregnancies.

This case highlights the importance of considering preeclampsia as a potential underlying cause of postpartum ischemic stroke. Early recognition of neurological symptoms and timely neuroimaging are critical to prevent severe maternal morbidity.

## Introduction

Preeclampsia has been identified as one of the major contributors to both maternal and perinatal morbidity worldwide [1]. It usually presents after 20 weeks of gestation, characterized by hypertension in combination with signs of multi-organ involvement, most commonly affecting the renal, liver, and central nervous systems. Although common symptoms include headache, visual disturbances, and epigastric pain, atypical presentations may occur and lead to severe complications [1]. Cerebrovascular accidents in pregnancy and the puerperium are rare but serious complications. They contribute to 4 to 11% of all maternal deaths [2]. Among all the cerebrovascular accidents, the occurrence of ischemic stroke (IS) in the context of preeclampsia is extremely rare but potentially life-threatening. It sometimes presents as the first manifestation of preeclampsia [3]. Here, the rare case of late-onset preeclampsia presenting with an acute ischemic stroke in the early puerperium has been highlighted.

## Case presentation

A 33-year-old woman, gravida 5 para 4 (two vaginal deliveries and two previous cesarean sections), with no significant medical history, underwent a cesarean delivery under spinal anesthesia. Her pregnancy had been regularly monitored, with no clinical or biological signs suggesting preeclampsia or other obstetric complications. The immediate postpartum period was initially uneventful.

Within 24 hours after delivery, she suddenly developed left-sided hemiparesis, left hemisensory loss, and left homonymous hemianopia, associated with conjugate gaze deviation to the right, warranting her transfer from the maternity unit to the Intensive Care Unit for management.

She was conscious but aphasic. Neurological examination revealed proportional left-sided hemiparesis, left-sided hemianesthesia, left-sided homonymous hemianopia, and rightward gaze deviation. Her vital signs showed hypertension (169/103 mmHg), tachycardia (113 bpm), respiratory rate of 16breaths/min, and SpO₂ at 97% on ambient air. Gynecologic examination revealed a well-contracted uterus, healthy lochia, and a clean cesarean wound.

Laboratory findings (Table 1) demonstrated moderate anemia with a Hemoglobin at 8.9 g/dL, marked leukocytosis (24.85 × 10⁹/L), normal platelet count (179 × 10⁹/L), and elevated lactate dehydrogenase (593U/L). Her liver function tests showed mildly elevated Aspartate transaminase (38 U/L) and normal Alanine aminotransferase (22 U/L). Her creatinine and urea were mildly elevated (0.87 mg/dL and 0.44 g/L, respectively). Coagulation profile was normal (prothrombin time at 100%, normal Partial thromboplastin time, fibrinogen levels at 2 g/L), but D-dimer levels were markedly increased (6.65 µg/mL). Her haptoglobin was at the low end of the normal range (0.68 g/L). Peripheral smear showed no significant abnormalities. 24-hour urine protein excretion was at 4.84 g/24h, confirming preeclampsia.

**Table 1:** Overview of laboratory findings

Biological data	On Admission	Normal range
Hemoglobin (g/dL)	8.9	12.5–17.5
White blood cells (×10⁹/L)	24.85	4.0–10.0
Platelets (×10⁹/L)	179	150–400
Creatinine (mg/dL)	0.87	0.7–1.2
Urea (g/L)	0.44	0.18–0.45
Aspartate Aminotransferase (U/L)	38	10–35
Alanine Aminotransferase (U/L)	22	8–35
Lactate dehydrogenase (U/L)	593	125–220
Prothrombin time (%)	100	70-100
Partial thromboplastin time (s)	30	25-39
Fibrinogen (g/L)	2	2-4
D-dimer (µg/mL)	6.65	<0.5
Haptoglobin (g/L)	0.68	0.5-2.2
24-hour urine protein excretion (g/24h)	4.84	<0.15

**Figure 1:**
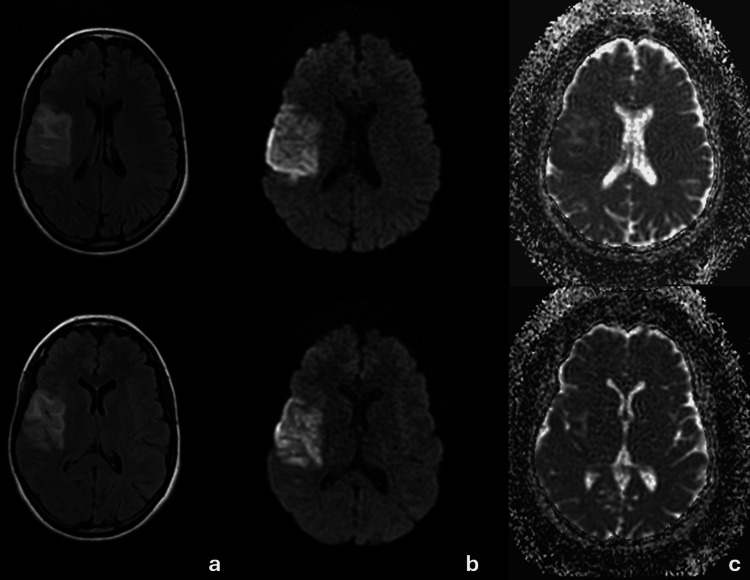
Brain MRI showing a cortico-subcortical infarct in the right fronto-parieto-insular region. (a) FLAIR sequence demonstrating early hyperintensity; (b) diffusion-weighted imaging showing hyperintensity; (c) apparent diffusion coefficient map demonstrating low signal, consistent with subacute ischemic infarction in the superficial territory of the right middle cerebral artery.

A brain MRI confirmed a subacute ischemic infarction involving the right fronto-parieto-insular cortico-subcortical regions, showing hyperintensity on diffusion-weighted imaging with partial pseudonormalization on the apparent diffusion coefficient (ADC) map and FLAIR hyperintensity, consistent with a subacute ischemic stroke. No hemorrhagic transformation was observed on T2* (T2-star-weighted gradient-echo) sequences (Figure 1).

Therapeutically, the patient was started on low-dose acetylsalicylic acid (75 mg/day) and levetiracetam 500 mg/12h. Blood pressure control was achieved with methyldopa 500 mg/8h and continuous intravenous perfusion of nicardipine initially. Cardiac evaluation and electrocardiogram were normal. Immunologic investigations (antinuclear antibodies, lupus anticoagulant, anticardiolipin antibodies) were negative.

The patient was transferred back to the maternity department 48 hours later with stable blood pressure. During hospitalization, she also underwent physiotherapy sessions aimed at improving muscle strength and preventing joint stiffness. She was discharged a week later on low-dose acetylsalicylic acid (75 mg/day) and Methyldopa (500 mg/8h).

Over the following six weeks, the patient experienced progressive and complete neurological recovery, with full recovery of motor function and independence in daily activities. Methyldopa was discontinued once blood pressure normalized. Prophylactic treatment with low-dose aspirin as well as low molecular weight heparin was recommended for future pregnancies.

## Discussion

Although less than 1% of women with preeclampsia experience a stroke during the postpartum period, preeclampsia and eclampsia remain among the major risk factors for both ischemic and hemorrhagic strokes in pregnancy [1,3].

In our patient, IS was the inaugural manifestation of late-onset preeclampsia, occurring within 24 hours after an otherwise uneventful, well-followed pregnancy. Its clinical presentation is similar to that in non-pregnant patients [4]. Neurological symptoms depend on the affected vascular territory, most commonly presenting with hemiparesis, cranial nerve palsies, or sensory deficits.

The peripartum and postpartum periods carry the highest risk for stroke, with an estimated incidence of 30 cases per 100,000 pregnancies [4,5]. Lanska and Kryscio [6] identified hypertensive disorders of pregnancy and cesarean delivery as the strongest risk factors for peripartum stroke.

The mechanisms linking preeclampsia to cerebral complications, including ischemic stroke, remain incompletely understood. Several mechanisms have been proposed, particularly endothelial dysfunction and disruption of the blood-brain barrier [7], which may impair cerebral autoregulation and alter cerebral perfusion pressure [8]. These alterations can lead to cerebral edema, microangiopathic injury, and vascular instability, ultimately predisposing to neurological complications such as IS [9].

Acute IS in the postpartum period requires careful evaluation of alternative etiologies beyond preeclampsia. Differential diagnoses included HELLP syndrome (hemolysis, elevated liver enzymes, and low platelet count), which was considered in our patient due to elevated LDH and low-normal haptoglobin. However, platelet count remained normal (179,000/mm³), and liver enzyme elevation was minimal, arguing against HELLP syndrome. Although laboratory findings suggested hemolysis, the absence of thrombocytopenia and the lack of schistocytes on peripheral smear made thrombotic microangiopathy (TMA) and microangiopathic hemolytic anemia (MAHA) diagnoses unlikely. As for the reversible cerebral vasoconstriction syndrome (RCVS), also an important postpartum differential diagnosis, it typically presents with thunderclap headache and segmental arterial narrowing on vascular imaging [4]. Our patient did not report such severe headaches, and the MRI pattern was consistent with a territorial middle cerebral artery infarction rather than multifocal vasoconstrictive changes, although the absence of dedicated vascular imaging (MRA) represents a limitation. Lastly, we also considered cerebral venous thrombosis (CVT), which typically involves venous infarcts, often hemorrhagic and not confined to a single arterial territory [4]. The MRI findings in our patient were consistent with arterial ischemia in the superficial MCA territory, making CVT unlikely.

In our patient, several findings supported the diagnosis of postpartum preeclampsia as the underlying cause of IS. These included the presence of new-onset severe hypertension in the immediate postpartum period, significant proteinuria (4.84 g/24 h), and laboratory evidence of hemolysis. Together, these findings fulfilled the diagnostic criteria for preeclampsia and supported its role as the precipitating factor for the cerebrovascular event.

Prompt diagnosis is crucial and relies on vascular neuroimaging, cardiac evaluation, and coagulation testing [10]. MRI is the imaging modality of choice, particularly diffusion-weighted and ADC sequences, which detect ischemic lesions in the hyperacute phase. CT may remain an acceptable alternative in resource-limited settings where MRI is not readily available [11].

Several case reports support the association between preeclampsia and IS. Grammatis et al. [12] described a 23-year-old primiparous woman at 35 weeks of gestation who presented with aphasia and hemiparesis due to a right MCA infarct. Farooque et al. [13] reported a postpartum IS successfully managed with antiplatelet therapy. Mnaili et al. [14] recently emphasized the importance of a multidisciplinary approach in managing IS during pregnancy to optimize maternal and fetal outcomes.

It is important to highlight the therapeutic dilemma of blood pressure management in the setting of acute ischemic stroke associated with preeclampsia. While permissive hypertension is generally tolerated in non-pregnant patients with acute ischemic stroke who are not candidates for thrombolysis [15], severe hypertension in preeclampsia requires prompt treatment to reduce the risk of intracerebral hemorrhage, eclampsia, and further endothelial injury [1]. Obstetric guidelines recommend lowering blood pressure to a target range of approximately 140-150 mmHg systolic and 90-100 mmHg diastolic, while avoiding abrupt reductions that could compromise cerebral perfusion [16].

In our patient, blood pressure was 169/103 mmHg, meeting criteria for treatment of severe hypertension in the context of preeclampsia. It should also be emphasized that although intravenous labetalol is commonly recommended as first-line therapy [17], its unavailability in our country led to the use of nicardipine, which is an effective and widely accepted alternative [18]. Oral methyldopa was subsequently introduced for maintenance therapy once blood pressure stabilized, allowing adequate blood pressure control while minimizing the risk of both hemorrhagic transformation and worsening cerebral ischemia. Throughout the follow-up, the patient showed no side effects related to Methyldopa, although it has been associated with postpartum depression [19], most likely due to its limited use in our case.

Women with a history of preeclampsia are at increased risk of recurrence in subsequent pregnancies as well as long-term cardiovascular complications [20]. Current guidelines recommend preventive strategies such as low-dose aspirin in future pregnancies, initiated early in gestation in women at high risk of preeclampsia [21]. In selected cases, particularly when thrombotic risk factors are present, additional measures such as low molecular weight heparin may be considered to reduce maternal complications [22].

## Conclusions

This case highlights IS as a rare yet potentially devastating complication of late-onset preeclampsia in the early postpartum period. Neurological symptoms may precede classical prodromal signs, underscoring the need for prompt recognition and early neuroimaging. In women with a history of preeclampsia complicated by IS, preventive strategies in subsequent pregnancies with low-dose aspirin and, when clinically indicated, low molecular weight heparin, should be individualized to reduce recurrence risk and optimize maternal outcomes.

## References

[1] Magee LA, Nicolaides KH, von Dadelszen P (2022). Preeclampsia. N Engl J Med.

[2] Yger M, Weisenburger-Lile D, Alamowitch S (2021). Cerebrovascular events during pregnancy and puerperium. Rev Neurol (Paris).

[3] Bushnell C, Chireau M (2011). Preeclampsia and stroke: Risks during and after pregnancy. Stroke Res Treat.

[4] Miller EC, Leffert L (2020). Stroke in pregnancy: A focused update. Anesth Analg.

[5] Karjalainen L, Tikkanen M, Rantanen K, Laivuori H, Gissler M, Ijäs P (2019). Pregnancy-associated stroke -a systematic review of subsequent pregnancies and maternal health. BMC Pregnancy Childbirth.

[6] Lanska DJ, Kryscio RJ (2000). Risk factors for peripartum and postpartum stroke and intracranial venous thrombosis. Stroke.

[7] McDermott M, Miller EC, Rundek T, Hurn PD, Bushnell CD (2018). Preeclampsia: Association with posterior reversible encephalopathy syndrome and stroke. Stroke.

[8] Belfort MA, Varner MW, Dizon-Townson DS, Grunewald C, Nisell H (2002). Cerebral perfusion pressure, and not cerebral blood flow, may be the critical determinant of intracranial injury in preeclampsia: a new hypothesis. Am J Obstet Gynecol.

[9] Hammer ES, Cipolla MJ (2015). Cerebrovascular dysfunction in preeclamptic pregnancies. Curr Hypertens Rep.

[10] Patil S, Rossi R, Jabrah D, Doyle K (2022). Detection, diagnosis and treatment of acute ischemic stroke: Current and future perspectives. Front Med Technol.

[11] Lackovic M, Nikolic D, Jankovic M, Rovcanin M, Mihajlovic S (2023). Stroke vs. preeclampsia: Dangerous liaisons of hypertension and pregnancy. Medicina (Kaunas).

[12] Grammatis AL, Catton HL, Hilton D (2019). Ischaemic stroke and pre-eclampsia in the third trimester of pregnancy: A diagnostic and therapeutic challenge. BMJ Case Rep.

[13] Farooque U, Cheema O, Karimi S, Pillai B, Liaquat MT (2020). Postpartum ischemic stroke: A rare case. Cureus.

[14] Mnaili MA (2023). Ischaemic stroke in pregnancy: Case report and review of literature. J Cerebrovasc Sci.

[15] Cantone M, Lanza G, Puglisi V (2021). Hypertensive crisis in acute cerebrovascular diseases presenting at the emergency department: A narrative review. Brain Sci.

[16] American College of Obstetricians and Gynecologists (2020). Gestational Hypertension and Preeclampsia: ACOG Practice Bulletin, Number 222. Obstet Gynecol.

[17] Magee LA, von Dadelszen P, Singer J (2016). The CHIPS randomized controlled trial (control of hypertension in pregnancy study): Is severe hypertension just an elevated blood pressure?. Hypertension.

[18] Bij de Weg JM, de Boer MA, Gravesteijn BY (2024). Optimal treatment for women with acute hypertension in pregnancy; a randomized trial comparing intravenous labetalol versus nicardipine. Pregnancy Hypertens.

[19] Wiciński M, Malinowski B, Puk O, Socha M, Słupski M (2020). Methyldopa as an inductor of postpartum depression and maternal blues: A review. Biomed Pharmacother.

[20] Coban U, Takmaz T, Unyeli OD, Ozdemir S (2021). Adverse outcomes of preeclampsia in previous and subsequent pregnancies and the risk of recurrence. Sisli Etfal Hastan Tip Bul.

[21] Alsulami FT, Hamed EM (2026). Early initiation of low-dose aspirin for the prevention of pre-eclampsia in high-risk pregnancies. Sci Rep.

[22] Baroutis D, Koukoumpanis K, Tzanis AA (2025). Low-molecular-weight heparin in preeclampsia: Effects on biomarkers and Prevention: A narrative review. Biomedicines.

